# Leptin inhibits proliferation of breast cancer cells at supraphysiological concentrations by inhibiting mitogen-activated protein kinase signaling

**DOI:** 10.3892/ol.2014.2085

**Published:** 2014-04-25

**Authors:** MICHAEL WEICHHAUS, JOHN BROOM, KLAUS WAHLE, GIOVANNA BERMANO

**Affiliations:** 1 Centre for Obesity Research and Epidemiology (CORE), Institute for Health and Welfare Research, Robert Gordon University, Aberdeen AB10 7GJ, UK; 2 Cancer Medicine Research Group, School of Medicine and Dentistry, University of Aberdeen, Medical School, Aberdeen AB25 2ZD, UK

**Keywords:** leptin, breast cancer, obesity, cell proliferation, mitogen-activated protein kinase cell signaling pathway

## Abstract

Leptin is a hormone secreted by white fat tissue and signals the amount of overall body fat to the hypothalamus. The circulating concentration of leptin correlates with the level of obesity. Breast cancer risk is higher in obese postmenopausal women compared with postmenopausal women of a normal weight, and high leptin concentrations may contribute to this risk. In the present study, SK-BR-3 and MDA-MB-231 breast cancer cell lines were treated with various concentrations (6.25–1,600 ng/ml) of recombinant leptin and changes in cell proliferation were assessed. The SK-BR-3 breast cancer cells exhibited a concentration-dependent increase in proliferation with physiological leptin concentrations (<100 ng/ml), but no further increase in proliferation at high leptin concentrations (>100 ng/ml) was observed. Cell proliferation was not affected at supraphysiological leptin concentrations (>800 ng/ml) in SK-BR-3 cells, whereas it decreased in MDA-MB-231 cells. Therefore, cell signaling and cell cycle changes were assessed at supraphysiological concentrations (1,600 ng/ml). In the two cell lines, leptin treatment decreased the mitogen-activated protein kinase (MAPK) cell signaling pathway activation. Leptin treatment did not increase Akt phosphorylation or significantly alter the cell population distribution across cell cycle stages. To the best of our knowledge, leptin-induced growth inhibition of breast cancer cells at supraphysiological concentrations has not been reported in the literature to date, and the findings of this study suggest that reduced MAPK activity may be the underlying cause. Thus, the effect of leptin on breast cancer growth warrants further investigation since leptin is considered to be one of the main mediators in the obesity-breast cancer connection.

## Introduction

Obesity, the accumulation of excess fat tissue, is a risk factor for the development of postmenopausal breast cancer (1). The molecular changes in metabolism associated with obesity are thought to contribute to this phenomenon (2). One of these molecular changes is increased circulating leptin levels in obese individuals (3). Leptin is a 16 kDa peptide hormone predominantly produced by white fat tissue (4). Its main function is to signal to the hypothalamus, which in response regulates satiety and energy expenditure (5). Notably, leptin is also responsible for the normal formation of the mammary gland in humans (6), suggesting an involvement in mammary tissue growth and differentiation, and potentially malignant transformation. Epidemiologically, leptin concentrations are higher in patients with breast cancer compared with healthy individuals, independent of body weight (7). Additionally, leptin receptor (Ob-R) expression is increased in breast tumor tissue compared with surrounding tissue (8). Breast cancer cell lines have also been found to express leptin and Ob-R (9).

Previous *in vitro* experiments investigating the effects of leptin treatment on cell proliferation and tumor growth have revealed conflicting results. In MDA-MB-231 breast cancer cells, leptin induced a robust concentration-dependent increase in proliferation in two independent studies (10,11). Conversely, in MCF-7 breast cancer cells, leptin treatment increased (12) and decreased (11) proliferation. Similarly, in human epidermal growth factor (HER)-2 overexpressing SK-BR-3 breast cancer cells, leptin treatment increased proliferation between 5 and 50 ng/ml, but not at 100 ng/ml (11). These controversial findings warrant the need for further investigation into the effects of leptin on proliferation in a human breast cancer *in vitro* cell system.

Notably, a number of studies investigating the effect of cytokines on proliferation changes in cell culture models used concentrations which are several-fold greater than the highest known physiological concentration, such as insulin treatment (13,14) or tumor necrosis factor-α treatment (15,16). However, previous studies investigating the effect of leptin treatment on proliferation in breast cancer cells (10,11,17) did not or only marginally exceeded maximal physiological leptin concentrations of 100 ng/ml (3). The highest leptin concentration used *in vitro* to examine changes in T47D breast cancer cell proliferation was 1,000 ng/ml. However, increased cell proliferation was only observed with up to 100 ng/ml leptin (18). Thus, while previous data appear to indicate a growth inhibitory effect of leptin at above physiological concentrations, it has not yet been fully investigated. Therefore, the present study aimed to explore the effects of supraphysiological leptin concentrations on proliferation in two breast cancer cell lines.

The present study aimed to investigate the effects of physiological and supraphysiological levels of leptin (≤1,600 ng/ml) on proliferation in SK-BR-3 and MDA-MB-231 breast cancer cells, two cell lines representative of HER-2-positive and basal-type breast cancer subtypes, respectively. The activation of phosphatidylinositide 3-kinase (PI3K) and mitogen-activated protein kinase (MAPK) cell signaling pathways and distribution across cell cycle stages were assessed in the two cell lines following treatment with 1,600 ng/ml leptin.

## Materials and methods

### Cell lines

SK-BR-3 and MDA-MB-231 breast cancer cell lines were purchased from the American Type Culture Collection (Manassas, VA, USA). The two cell lines were routinely cultured in RPMI-1640 medium (including 25 mM HEPES, 1× Glutamax™; Gibco, Paisley, UK) supplemented with 10% fetal calf serum (FCS; Pierce Biosciences, Cramlington, UK), 100 U/ml penicillin and 100 μg/ml streptomycin (Gibco).

### Bromodeoxyuridine (BrdU) proliferation assay

Cell proliferation was detected using the Proliferation ELISA kit (Roche Diagnostics GmbH, Penzberg, Germany). The two cell lines were plated at a density of 5×10^3^ cells/well in 96-well plates with 100 μl/well growth medium and incubated for 24 h at 37°C. Cells were starved for 24 h in RPMI-1640 medium without FCS supplementation, and then treated for 24 or 48 h with 6.25–1,600 ng/ml leptin in replicates of six in starvation medium. During treatment, the medium was supplemented with 10 μM BrdU. Cell proliferation was assessed as previously described (16). The experiment was repeated for a total of three independent times. Each experiment had six replicates for each leptin concentration.

### PI3K and MAPK phosphorylation ELISA

Cell-based ELISA Phospho-Akt (S473) Immunoassay and Phospho-extracellular signal-regulated kinase (ERK)1/ERK2 (T202/Y204) Immunoassay were purchased from R&D Systems (Abingdon, UK). The cells were plated in a supplied clear bottom, black-walled, 96-well plate at a density of 5×10^3^ cells/well with 100 μl/well growth medium, and incubated for 24 h at 37°C. The cells were starved for 24 h as mentioned above and then treated with 1,600 ng/ml leptin for 5–20 min in duplicates. Phosphorylation of protein kinase B (Akt) or ERK1/2 was then assessed as previously described (16). The experiment was repeated for a total of three independent experiments with two replicates for each time point in each experiment.

### Cell cycle analysis

Changes in the cell distribution across cell cycle stages were assessed by measuring the DNA content in cells using flow cytometry following leptin treatment. The DNA-specific dye was propidium iodide (PI; Sigma-Aldrich, Gillingham, UK). The cells were plated at a density of 5×10^5^ cells/well in six-well plates with 3 ml growth medium, and incubated for 24 h at 37°C. The cells were starved for 24 h, treated with 1,600 ng/ml leptin for 24 h and then harvested, treated and analyzed as described previously (16).

### Statistical analysis

The findings were analyzed for statistical significance using univariate analysis of variance between the control and each treatment concentration for cell proliferation analysis and between the control and each time point in the cell signaling pathway analysis, followed by Dunnett’s post hoc t-tests. Differences in the distribution across cell cycle stages between the control and leptin-treated cells were assessed for each cell cycle stage using Student’s t-test. P<0.05 was considered to indicate a statistically significant difference.

## Results

### Changes in cell proliferation following leptin treatment

**Figure 1:**
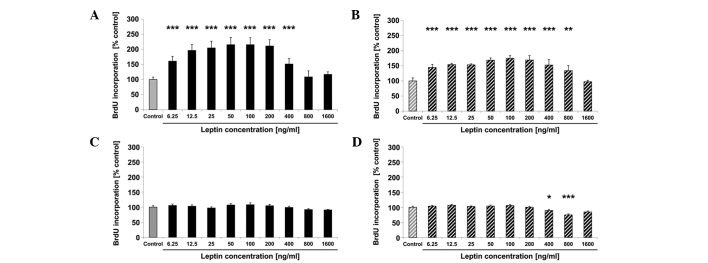
Changes in cell proliferation following treatment of (A and B) SK-BR-3 and (C and D) MDA-MB-231 breast cancer cells for (A and C) 24 h and (B and D) 48 h, with a range of leptin concentrations. Bars represent BrdU incorporation in relation to the respective control within each graph, and are expressed as a percentage of the control. Error bars represent standard error of the mean of two experiments, each consisting of six replicates, i.e., 12 data points for each bar. Significance was determined using Dunnett’s post hoc t-test following one-way analysis of variance (^*^P<0.05, ^**^0.01<P<0.001 and ^***^P<0.001, vs. the control). BrdU, bromodeoxyuridine

In the SK-BR-3 cells, proliferation increased by 61, 96, 104, 115, 115, 110 and 51% following treatment with 6.25, 12.5, 25, 50, 100, 200 and 400 ng/ml leptin, respectively, for 24 h compared with the untreated control (all P<0.001) (Fig. 1A). There was no significant difference in cell proliferation between the untreated cells and cells treated with 800 or 1,600 ng/ml leptin for 24 h (Fig. 1A). After 48 h of treatment, cell proliferation increased significantly by 44, 53, 53, 69, 75, 69, 52 and 33% following treatment with 6.25, 12.5, 25, 50, 100, 200, 400 ng/ml (all P<0.001) and 800 ng/ml (P=0.009) leptin, respectively compared with the untreated control (Fig. 1B). There was no change in proliferation after 48 h of treatment with 1,600 ng/ml leptin (Fig. 1B). In MDA-MB-231 breast cancer cells, proliferation did not change significantly after 24 h of treatment with leptin (Fig. 1C) and decreased significantly by 11% (P=0.023) and 26% (P<0.001) after 48 h of treatment with 400 and 800 ng/ml (P<0.001) of leptin, respectively, compared with the untreated control (Fig. 1D). Based on the findings obtained on growth inhibition following treatment with 1,600 ng/ml leptin, cell signaling and cell cycle changes were assessed to determine the underlying mechanisms responsible for growth inhibition in the two breast cancer cell lines.

### Changes in PI3K and MAPK cell signaling pathway activity following leptin treatment

**Figure 2:**
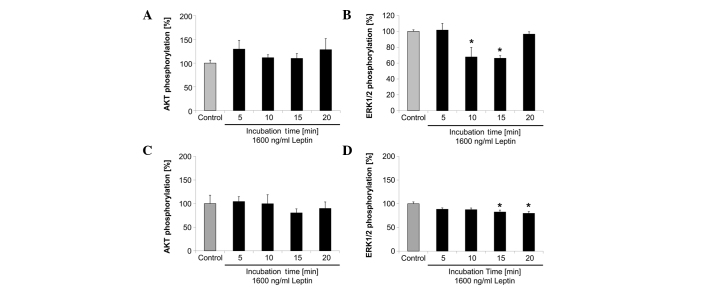
Changes in (A and C) Akt phosphorylation and (B and D) ERK1/2 phosphorylation as indicators of changes in the PI3K or MAPK cell signaling pathway, respectively, following 1,600 ng/ml leptin treatment of (A and B) SK-BR-3 and (C and D) MDA-MB-231 breast cancer cells for the indicated time periods. Bars represent Akt or ERK1/2-phosphorylation in relation to their respective control within each graph, and are expressed as a percentage of the control. Error bars represent standard error of the mean of three experiments, each consisting of two replicates, i.e., six data points for each bar. Significance was obtained using Dunnett’s post hoc t-test following univariate analysis of variance (^*^P<0.05, vs. the control). ERK, extracellular signal-regulated kinase; PI3K, phosphatidylinositide-3 kinase; MAPK, mitogen-activated protein kinase.

In the SK-BR-3 cells, Akt-phosphorylation did not change significantly following treatment with 1,600 ng/ml leptin for up to 20 min compared with the control (Fig. 2A). ERK1/2-phosphorylation decreased by 32 and 34% after 10 (P<0.001) and 15 min (P<0.001) of treatment with 1,600 ng/ml leptin, respectively, compared with the control (Fig. 2B). In the MDA-MB-231 cells, Akt-phosphorylation did not change significantly following treatment with 1,600 ng/ml leptin (Fig. 2C), whereas ERK1/2-phosphorylation decreased significantly by 17 and 20% after 15 (P=0.026) and 20 min (P=0.011) of treatment with 1,600 ng/ml leptin, respectively, compared with the control (Fig. 2D).

### Changes in distribution of cell population across cell cycle stages

In the SK-BR-3 cells, 1,600 ng/ml leptin treatment may increase the G_1_-phase population and decrease the G_2_-phase population (Table I). Cells in the G_1_-phase increased by 4.5 percentage points (11% increase) and cells in the G_2_-phase decreased by 2.0 percentage points (12% decrease). In MDA-MB-231 cells, the subG_1_-phase population increased by 1.2 percentage points (7% increase) and the G_1_-phase population decreased by 1.0 percentage point (2% decrease) following treatment with 1,600 ng/ml leptin (Table I). None of the observed changes were significantly different.

## Discussion

Previous *in vitro* studies have demonstrated that leptin induces cell proliferation in a variety of breast cancer cell types, within physiological concentrations (25–100 ng/ml) (6,9–12,18). Conversely, the same studies did not observe increased cell proliferation with leptin concentrations exceeding 100 ng/ml. The findings of the present study confirmed the increased cell proliferation in SK-BR-3 breast cancer cells, but not in MDA-MB-231 cells at physiological concentrations. Furthermore, leptin treatment at supraphysiological concentrations did not increase cell proliferation in the SK-BR-3 cells, but inhibited proliferation of the MDA-MB-231 cells. To the best of our knowledge, this study was the first to indicate the potential of leptin treatment to inhibit cell proliferation in breast cancer cells. The mechanism by which supraphysiological leptin concentrations induce growth inhibition may involve decreased activation of the Ras-mediated MAPK pathway.

As a potential explanation, leptin may interact with the HER-2/neu receptor in SK-BR-3 breast cancer cells, which is overexpressed in these cells, resulting in decreased MAPK activity. Soma *et al* reported that SK-BR-3 cells treated with leptin (500 ng/ml) resulted in increased phosphorylation of the HER-2/neu receptor (20). This cross-talk was identified as being responsible for an increase in ERK1/2 phosphorylation, which was also observed following leptin treatment. The findings of the present study suggest that at higher leptin concentrations, this effect is inhibited. This may either be by high leptin levels inhibiting the potential of HER-2/neu to activate ERK1/2 or by inhibiting ERK1/2 phosphorylation directly. HER-2/neu and Ob-R transduct their proliferative signal through the PI3K and/or MAPK pathways (21), suggesting there may be an interaction on the targets downstream of the two receptors. Thus, leptin may have at least two modes of action, which appear to be antagonistic. First, leptin increases phosphorylation of HER-2/neu, which results in increased proliferation of SK-BR-3 breast cancer cells; second, leptin inhibits ERK1/2 phosphorylation, which is predominant at high leptin concentrations, thereby reducing the effect of increased HER-2/neu signaling.

In MDA-MB-231 breast cancer cells, which are HER-2-negative, interplay with HER-2/neu cannot account for the observed reduction in proliferation, indicating that growth inhibition at supraphysiological leptin concentrations is HER-2/neu independent. In a study aiming to potentiate the antitumor effects of cAMP-agonists, leptin induced apoptosis in MDA-MB-231 breast cancer cells when cAMP levels were increased (22), which resulted in ERK1/2 inactivation and the subsequent inhibition of protein kinase A (PKA) expression (23). Thus, at supraphysiological concentrations, leptin may not require elevated cAMP levels to decrease PKA, and this may provide a mechanism for the effects observed in our study.

These findings suggest that leptin exerts a biphasic effect on cell proliferation in SK-BR-3 breast cancer cells and that leptin signaling may play a role in breast cancer development and progression. Therefore, the inhibition of leptin signaling may be relevant for breast cancer prevention, particularly for obese individuals showing high levels of leptin and occurrence of breast cancer. Nutritional interventions (24) or anti-leptin treatment (25) may be considered as a potential preventative strategy and treatment for HER-2/neu overexpressing breast tumors, respectively. Further investigations into the inhibitory effects of leptin at high concentrations may reveal the unknown mechanisms in the connection between obesity and postmenopausal breast cancer.

## Figures and Tables

**Table I:** Changes of cell population distribution across cell cycle stages after 24 h of treatment with 1,600 ng/ml leptin.

	SK-BR-3 cells			MDA-MB-231 cells		
				
Cell cycle stage (%)	Control	Leptin-treated	P-value	Control	Leptin-treated	P-value
SubG_1_	20.56±3.01	20.4±1.68	0.2189	17.00±1.07	18.15±1.33	0.5077
G_0_/G_1_	39.90±2.00	44.385±2.10	0.1490	55.12±0.97	54.14±0.99	0.3830
S	6.30±0.45	5.86±0.81	0.6444	7.23±0.65	6.96±0.62	0.7677
G_2_	15.98±0.70	14.03±0.87	0.0863	12.88±0.43	12.71±0.48	0.4257

Values represent the mean ± standard error of three independent experiments. P-values were determined by Student’s t-test. Each experiment had two replicates, i.e., six data points for control and treatment.
